# Subclinically Anxious Adolescents Do Not Display Attention Biases When Processing Emotional Faces – An Eye-Tracking Study

**DOI:** 10.3389/fpsyg.2018.01584

**Published:** 2018-08-24

**Authors:** Kathrin Cohen Kadosh, Simone P. Haller, Lena Schliephake, Mihaela Duta, Gaia Scerif, Jennifer Y. F. Lau

**Affiliations:** ^1^Department of Experimental Psychology, University of Oxford, Oxford, United Kingdom; ^2^School of Psychology, University of Surrey, Guildford, United Kingdom; ^3^Research Group Neural Mechanisms of Human Communication, Max Planck Institute for Human Cognitive and Brain Sciences, Leipzig, Germany; ^4^International Max Planck Research School on Neuroscience of Communication: Function, Structure, and Plasticity, Leipzig, Germany; ^5^Department of Psychology, Institute of Psychiatry, King’s College London, London, United Kingdom

**Keywords:** adolescence, anxiety, emotion processing, eye-tracking, individual differences, pupil dilation

## Abstract

Recent research suggests that early difficulties with emotion regulation go along with an increased risk for developing psychiatric disorders, such as anxiety disorders for example. Adolescent anxiety is often referred to as a gateway disorder, due to its high predictability for lifelong persistent mental health problems. It has been shown that clinically anxious adolescents exhibit attention biases toward negative stimuli, yet whether these biases can also be found in the subclinical range of subclinically anxious adolescents is currently unclear. In this study, we set out to investigate this question by combining an emotional Go-Nogo task with eye-tracking techniques to assess attention biases for emotional faces in a subclinical sample of 23 subclinically anxious adolescent girls. This combined approach allowed us to look at both, behavioral and covert attention biases. Using both traditional and Bayesian hypothesis testing, we found no evidence for a bias toward negative, threat-relevant stimuli in the behavioral level or eye-tracking data. We believe that the results can help close a gap in the current literature by showing that like low-anxious adolescents, subclinically anxious adolescents do not exhibit attention biases when viewing de-contextualized emotional stimuli in the Overlap task. Together with previous research findings in clinically anxious participants which have reported high levels of attention biases, our results seem to suggest that attention biases do no increase linearly as a function of individual anxiety level. Future research is now needed to explore the contribution of additional factors, such as depression for example.

## Introduction

Adolescence is a transitional developmental period, spanning the years between the onset of puberty and adulthood. This period is marked by distinct changes in emotional behavior (Moor et al., 2010; Sebastian et al., 2010; Guyer et al., 2012) with some suggestion that early difficulties with processing and regulating emotions increase risk for later psychiatric problems. Indeed, with approximately 1 in 4 adolescents exhibiting increased levels of worry and anxiety (Kessler et al., 2005; Keshavan et al., 2014), these adolescent-onset difficulties may act as a gateway disorder to lifelong persistent mental health problems (Kessler et al., 2007; Keshavan et al., 2014). Gaining a better understanding how early difficulties in emotion regulation contribute to the development of anxiety could also help with designing new, adjunct interventions for supplementing current frontline treatments, such as CBT for example. This is important as recent research has highlighted how intervening with sub-threshold symptoms in adolescents can be effective in reducing the risk of full-syndrome depression (Garber et al., 2009; Brent et al., 2015).

In terms of individual differences, most research that looked at how difficulties in processing and regulating emotions relate to anxiety has focused on the role of cognitive biases. Cognitive biases, such as increased attention toward threat are thought to maintain or even precipitate anxiety (Bar-Haim, 2010). Data show that both anxious adults and children seem to orient more toward threat-relevant stimuli (Bar-Haim et al., 2007; Shechner et al., 2012; Dudeney et al., 2015). At the sub-threshold level, however, the findings are much less clear. For example, research has shown that non-clinically anxious child participants exhibit both, orientation away from threat or no difference at all (Bar-Haim et al., 2007; Shechner et al., 2012). This seems to suggest that the relationship between anxiety and attention biases is not a straightforward, linear one, with increasing anxiety levels automatically linked to biases of higher magnitude. It also casts doubt on the suitability of attention bias modification paradigms for preventive interventions in this group. This is particularly problematic for highly anxious children and adolescents who do not yet meet clinical threshold but are nonetheless at an increased risk for progressing to an anxiety disorder in the future. Gaining a better understanding of how individual variations in anxiety levels contribute to attention biases in this population is important if we want to find early markers of atypical development for targeted intervention approaches (Haller et al., 2013).

One reason for the lack of clear results in subclinical groups of anxious participants may be methodological issues. Up until now, bias research has relied heavily on the *dot-probe paradigm* (MacLeod et al., 1986), where two stimuli (often faces) are shown simultaneously (one threat-relevant stimulus and one neutral stimulus), followed by a small dot-probe in the location just occupied by one of them. A bias toward threat is then inferred if participants are faster to respond to a dot that is presented in same location as the threat-relevant stimulus. With regards to the ambiguous findings in non-clinically anxious participants, one explanation might be that the dot-probe measure of differences in attention shifting patterns is not sensitive enough to uncover subtle anxiety in these pre-clinical populations. In a recent study, Cohen Kadosh et al. (2014) used the Overlap task (Bindemann et al., 2005) to assess how differences in trait anxiety affect the processing of emotional face stimuli during development. The Overlap task is an emotional Go-No-Go task, where participants focus on a central go/no-go signal, before, on go trials, responding to a peripheral line target. The Overlap task varies from the dot-probe task in that participants are required to focus on a central emotional stimulus before disengaging attention to make an overt attention shift toward a neutral stimulus. The use of a central rather than two peripheral faces with emotional expressions also allows us to assess the effects of emotional expressions more directly, as participants need to actually look at one face rather than distributing their attention between two faces simultaneously. Cohen Kadosh et al. (2014) found that a group of late adolescents (aged17–18 years) processed the different emotional expressions fear, happiness and neutral at comparable speed and accuracies in this task.

For the current study, we wanted to extend on these previous studies using eye-tracking techniques with the Overlap task. Eye-tracking techniques record eye-movements and pupil dilation during stimulus presentation, with differences in either indices taken to reflect variations in attentional processing (Just and Carpenter, 1976). This technique provides an elegant approach toward a better understanding of the exact time course of attention allocation. It also provides some insight into whether participants actually process the emotion information differentially at the pre-disengagement stage from the centrally presented face. So far, only five studies have used eye-tracking techniques to understand viewing patterns in clinically anxious children and adolescents. Across these studies, results have been mixed, as can be seen in **Table 1A** (see also **Table 1B** for full details). For example, Shechner et al. (2013) found that anxious youth exhibited an early bias toward angry faces in comparison to non-anxious youth (mean age 12 years) in a passive dot-probe task. Similarly, In-Albon et al. (2010) found that 10-year-old children with separation anxiety disorder exhibited increased fixation time toward threat-relevant pictures, as did Seefeldt et al. (2014) for their sample of children (aged 7–14 years) with social phobia. Evidence for threat avoidance on the other hand was found by Gamble and Rapee (2009) with anxious adolescents (mean age 14 years) but not children (mean age 11 years) consistently moving the first fixation away from the angry faces. Last, Shechner et al. (2015) found evidence of threat avoidance in a visual scene task, which did not vary as a function of age across a sample of anxious children, adolescents and adults.

**Table 1A:** Overview of previous developmental attention bias studies that used eye-tracking techniques in children, adolescents and adults with different levels of anxiety. Shaded cells are coded as follows: gray = no bias found; green = bias towards threat; red = bias away from threat. Note the gap in research into subclinical levels of high anxiety across all age ranges.

Anxiety/Age	Low				High				Clinical		
7–12 years	3a,b,c‡								2c,d	3a,b,e	1d
12–18 years	1c								1d	1e	2c,d
19 + years	d										1d

*^a^In-Albon et al., 2010; ^b^Seefeldt et al., 2014; ^c^Gamble and Rapee, 2009; ^d^Shechner et al., 2015; ^e^Shechner et al., 2013.*

Also, as evident from **Tables 1A,B**, no research up until now has used eye-tracking techniques to investigate attention biases in subclinical samples of anxious youth. The present study aimed to address this gap. Testing a subclinical sample of anxious adolescents, we focused on two specific hypotheses: (1) We expected that both looking times and pupil dilation would vary as a function of emotional valence. Specifically, we expected to see longer scanning of fearful as opposed to happy or neutral faces, along with increased pupil dilation for fearful faces (a behavioral pattern which would be taken to reflect attention biases toward threatening stimuli). (2) We also expected that these effects would vary as a function of individual differences in trait anxiety, with increased scanning/dilation scores correlating with higher anxiety scores.

**Table 2:** Detailed information on studies listed in **(A)**. M_A_, Mean for Anxious Group; M_NA_, Mean for Non-Anxious Group; M_X/C_, Children; M_X/A_, Adolescents; RCMAS-C, Reynolds Children’s Manifest Anxiety Scale, Child version; RCMAS-P, Reynolds Children’s Manifest Anxiety Scale, Parent version; CDI, Children’s Depression Inventory; K-SADS-PL, Kiddie Schedule for Affective Disorders - Present and Lifetime version; SAI, Separation Anxiety Inventory, Child and Parent version; SASC, Social Anxiety Scale for Children; SCARED, Self-report for Childhood Anxiety Related Emotional Disorder; SCAS, Spence Children’s Anxiety Scale; SDQ, Strengths and Difficulties Questionnaire; SCASp, Spence Children’s Anxiety Scale (Parent Version); SDQp, Strengths and Difficulties Questionnaire (Parent Version); SPAI-C, Social Phobia and Anxiety Inventory for Children.

Reference	Participants (n)	Age (in years)	Gender	Paradigm and stimulus Type	Presentation Times	Anxiety Measure and Score	Effect Size
^a^In-Albon et al., 2010	23 separation anxiety disorder	*M* = 9.91 *SD* = 1.44	13 F 10 M	Separating and reuniting photographs:	4,000 ms	SAI-C: M_A_ = 22.9 M_NA_ = 8.1 SAI-P:M_A_ = 26.3 M_NA_ = 7.8	Medium effect size (Cohen’s *d* = 0.50)
		Range = 8–13		images of a woman separating from a child as threat stimuli		RCMAS-C: M_A_ = 11.7 M_NA_ = 8.1	
	17 non-anxious controls	*M* = 10.29 *SD* = 1.40 Range = 8–13	6 F 11 M	images of a woman reuniting with a child as potent non-threat stimuli		RCMAS-P: M_A_ = 13.1 M_NA_ = 5.5	
						CDI: M_A_ = 10.8 M_NA_ = 7.9	
^b^Seefeldt et al., 2014	30 social phobia	*M* = 9.9 *SD* = 1.37 Range = 8–12	13 F 17 M	Four types of picture pairs: happy face – neutral face angry face – neutral face	3,000 ms	SPAI-C: M_A_ = 20.7 M_NA_ = 3.8	Low to high effect sizes (Cohen’s d = 0.00 to Cohen’s d = 0.82)
	43 controls	*M* = 9.9	19 F	angry face – happy face		SASC: M_A_ = 46.1 M_NA_ = 26.7	
		*SD* = 1.4	24 M	neutral face – house (non-social control)			
		Range = 8–12					
^c^Gamble and Rapee, 2009	43 anxiety disorder:			Two types of picture pairs:	3,000 ms vs. 500 ms	SCAS: M_A/C_ = 33.1 M_NA/C_ = 13.1	Not specified
	- 19 children	*M* = 9.82 *SD* = 1.12 Range = 7–11	10 F 9 M	negative facial expression (anger, disgust, fear and sadness) - neutral facial expression		M_A/A_ = 35.0 M_NA/A_ = 12.3	
	- 24 adolescents	*M* = 13.99 *SD* = 1.4 Range = 12–17	11 F 13 M	happy facial expression – neutral facial expression		SDQ: M_A/C_ = 13.9 M_NA/C_ = 8.4 M_A/A_ = 12.7 M_NA/A_ = 7.6	
	49 controls:						
	- 20 children	*M* = 10.64	8 F			SCASp: M_A/C_ = 31.9 M_NA/C_ = 8.0	
		*SD* = 0.98	12 M			M_A/A_ = 33.8 M_NA/A_ = 8.0	
		Range = 8–11				SDQp: M_A/C_ = 13.4 M_NA/C_ = 4.9	
	- 29 adolescents	*M* = 13.65	14 F			M_A/A_ = 14.6 M_NA/A_ = 4.8	
		*SD* = 1.0	15 M				
		Range = 12–16					
^d^Shechner et al., 2015	19 anxiety disorder	*M* = 12.08 *SD* = 2.93 Range = 8–17	9 F 10 M	Visual scene task: central neutral image flanked by two threatening or two rewarding stimuli	5,000 ms	Self-reported SCARED: M_A_ = 25.26 M_NA_ = 9.82 Parent-reported SCARED: M_A_ = 36.79 M_NA_ = 4.82	Not specified
	26 healthy youth	*M* = 13.03	11 F				
		*SD* = 2.87	15 M				
		Range = 8–17					
^e^Shechner et al., 2013	18 anxiety disorder	*M* = 12.25 *SD* = 3.27 Range = 8–17	9 F 9 M	Three types of picture pairs:	10,000 ms	Diagnoses by K-SADS-PL (no measure of current anxiety described)	Not specified
				angry face - neutral face			
				happy face - neutral face			
				neutral face - neutral face			
	15 non-anxious youth	*M* = 14.31	10 F				
		*SD* = 2.23	5 M				
		Range = 8–17					

## Materials and Methods

### Participants

Twenty three adolescent girls^1^ were selected to participate in the current study (mean age: 15.43, SD: 1.53 years, range 12–18 years). All participants completed the State Trait Anxiety Questionnaire for Children (STAIC) before doing the behavioral task. The STAIC features two self-report scales for measuring distinct anxiety concepts of trait anxiety. Both anxiety scales consist of 20 trait/state anxiety statements that ask participants to indicate on a scale from 1 to 3 how they feel in general (trait) or in the current moment (state) (1 being rarely anxious and 3 being often anxious). We note that the STAIC is not a clinical assessment tool and therefore no cut-offs exist. For the current sample, the mean trait anxiety level of 39 points (SD: 8.7) was equivalent of the 92nd percentile (Spielberger et al., 1997)^2^, and mean state anxiety level of 31 points (SD: 4.4) was equivalent to the 57th percentile.

Participants self-reported no history of psychiatric illness or learning difficulty. Informed written consent was obtained from the participant’s primary caregiver, and informed written assent was obtained from all participants prior to testing. The study was approved by the local ethics committee at the University of Oxford (MSD/IDREC/C2/2012/12 ‘Anxiety based differences in adolescent fear learning’).

### Stimuli

For the Overlap task (Bindemann et al., 2005; Cohen Kadosh et al., 2014), we created a stimulus set from 9 color photographs of female faces, 3 women × 3 emotional expressions (fearful, neutral, happy), selected from the NimStim set (Tottenham et al., 2009). All pictures were cropped to show the face in frontal view and to exclude the neck and haircut of the person. For the face + target stimuli, a fixation cross was superimposed onto the face between the two eyes, and two black peripheral lines were presented on each side of the face. In total, 36 different stimuli [3 women × 3 expressions × target right or left of the face × green/red fixation cross (go/no-go trials)] were created. When viewed at a distance of approximately 57 cm, the three faces subtended 9.8° × 10° of visual angle. The two lines were presented at 22°of visual angle, subtending 0.2° × 2° of visual angle. Note that we used only female faces in the current study in order to keep any task-irrelevant stimulus variation at a minimum.

### Eye-Tracking Apparatus

During the Overlap task, eye movements were recorded using a Tobii TX300, collecting gaze data at 300 Hz. All calibration and task stimuli were presented using a custom-made code using MATLAB 2012a (The Math Works Inc., Natick, MA, United States) and Psychophysics toolbox^3^. Calibration consisted of the presentation of a star-shaped calibration target at 9 points of the display (20%, 50%, and 80% of both horizontal and vertical span). Stimuli were presented on a 24-in monitor with a spatial resolution of 1920 × 1080 pixels, run at a 60 Hz refresh rate.

### Procedure

**FIGURE 1:**
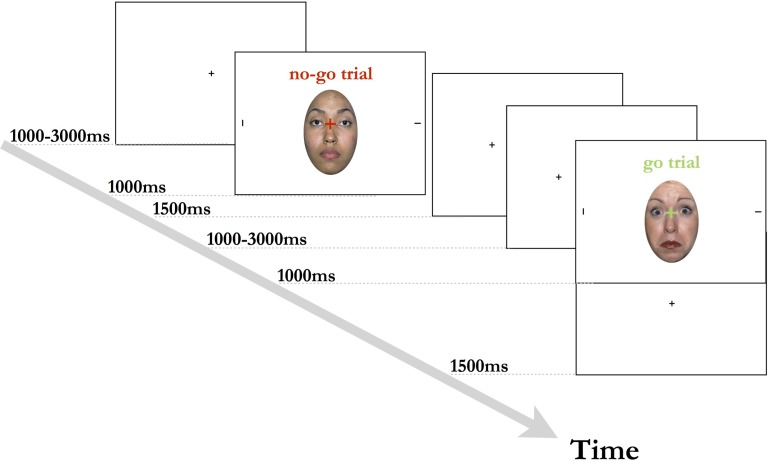
Experimental design used in the current study. Stimulus shown is from the NimStim stimulus collection of facial expression (Tottenham et al., 2009) and consent for publication has been provided under CC license.

The experimental session took place in a slightly dimmed, soundproof room. Prior to the start of the task, the participants’ eye movements were calibrated and task instructions were presented. Participants were seated approximately 57 cm away from the computer monitor, and were instructed to focus on the center fixation cross when it appeared on the screen. Each trial began with a central black fixation cross on a white background, being presented for 1000 ms. The fixation cross was then replaced for 500 ms by the face + target stimulus, with a red or green fixation cross super-imposed onto a face flanked by two peripheral black lines. The color of the fixation cross indicated whether the trial was a go trial (green color) or a no-go trial (red color). During the go trials, the participant’s task was to indicate which of the two lines on either side of the face was presented horizontally. Participants were instructed to indicate the location of the target stimulus via a button press on a keyboard. During no-go trials, participants were instructed not to respond and to wait for the next trial to begin. The face + target stimulus was followed by a blank screen with black fixation cross, which was displayed for 2000–4000 ms, or until a response was registered (see also **Figure 1**). Each session began with 12 practice trials (6 go trials, 6 no-go trials), with each emotional expression being shown 4 times. The practice was followed by 4 blocks of 36 trials with a ratio of 2:1 go (24) to no-go (12) trials, with each facial expression (fearful/neutral/happy) being shown an equal number of times in the trials. Additionally, we created three pseudo-randomized variations of the task to ensure that each emotional expression and trial type varied systematically throughout the blocks. Participants were encouraged to take self-paced breaks in-between testing blocks.

### Eye-Tracking Data Reduction: Invalid Data and Trials

Eye-tracking data (gaze data and pupil diameter of the left eye) was processed using custom-made MATLAB scripts. Specifically, data recorded from fixation onset for 1500 ms was used in the analysis. Gaze and pupil data was included in the analysis if the eye-tracker successfully tracked both eyes, the recorded gaze was within screen area and the pupil diameter was less than 5 mm. Blinks were detected as an instantaneous rate of change of pupil diameter greater than 0.1 mm for both eyes. For the duration of the blinks the gaze data was replaced with the last good recorded value, while the pupil diameter data was linearly interpolated. For the pre-processing of the pupil dilation data, a median filter (smoothing filter) was applied over 100 ms (33 samples) running windows of the pupil diameter data and detected blinks were linearly interpolated with respect to the values just before and after each identified blink. Pupil diameter was baseline corrected with respect to the mean of over 200 ms prior to stimulus onset. Mean area amplitudes were calculated for the time window of 500–1500 ms post stimulus presentation.

## Results

### Behavioral Results

Mean reaction times (RTs) were calculated for correct go trials only [mean RTs/standard deviation: fearful = 675 ms/79 ms; happy = 678 ms/76 ms; neutral = 677 ms/69 ms]. These were subjected to a one-way repeated-measures ANOVA with the within-subject factor “expression” (fearful, neutral, happy). The main effect of expression was not significant [*F*(2,44) = 0.116, *p* = 0.851, $\eta_{\text{p}}^{2}$ = 0.005]. We also conducted Bayesian analysis to obtain a clearer picture of whether the null hypothesis would be the better representation of the data (Wagenmakers, 2007; Masson, 2011; Jarosz and Wiley, 2014) and the Bayesian Factors of *BF01* = 21.6 suggests that the data are 21.6 times more likely to be observed under the null hypothesis which predicted no significant differences between the three emotional expressions. Further, when trait anxiety was included as a covariate in the analysis, neither main effect of emotional expression, nor the interaction between emotion expression × trait anxiety was significant (main effect of expression: [*F*(2,42) = 1.02, *p* = 0.360, $\eta_{\text{p}}^{2}$ = 0.046]; interaction emotional expression × trait anxiety [*F*(2,42) = 0.925, *p* = 0.394, $\eta_{\text{p}}^{2}$ = 0.042]; *BF01* = 13.3).

For the accuracy rates [mean accuracy rates/standard deviation: fearful = 98%/0.30%; happy = 98%/0.30%; neutral = 94%/0.23%], the main effect of expression was significant [*F*(2,44) = 27.7, *p* < 0.001, $\eta_{\text{p}}^{2}$ = 0.558; *BF01* < 0.001]. Further analysis showed that this was due to a significant decrease in accuracy rates for trials with a neutral expression (neutral face vs. fearful face: *t*(22) = 6.0, *p* < 0.001, CI%[0.026,0.053]; neutral face vs. happy face: *t*(22) = 10.0, *p* < 0.001, CI%[0.030,0.050]; fearful vs. happy face: *t*(31) = 0.125, *p* = 0.901, CI%[-0.014,0.016]). Interestingly, when trait anxiety was included as a covariate in the analysis, neither main effect of emotional expression, nor the interaction between emotion expression × trait anxiety was significant (main effect of expression: [*F*(2,48) = 2.12, *p* = 0.145, $\eta_{\text{p}}^{2}$ = 0.092]; interaction emotional expression × trait anxiety [*F*(2,48) = 1.10, *p* = 0.331, $\eta_{\text{p}}^{2}$ = 0.050]; *BF01* = 12.3).

### Viewing Patterns for the Three Emotional Expressions

**FIGURE 2:**
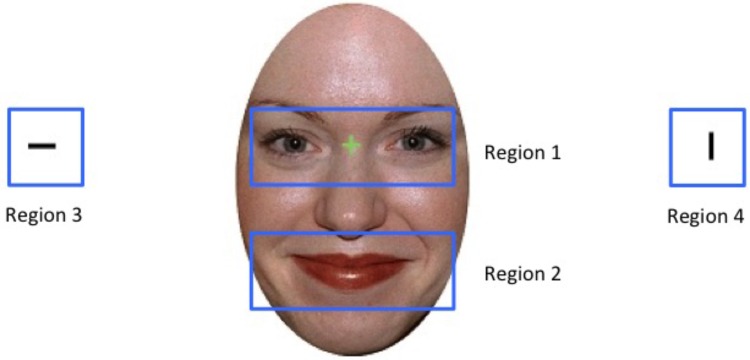
Depiction of the four eye-tracking regions for an example stimulus. Stimulus shown is from the NimStim stimulus collection of facial expression (Tottenham et al., 2009) and consent for publication has been provided has been provided under CC license.

We then investigated whether participants’ viewing patterns varied as a function of the emotional expression displayed. Specifically, we divided the display into four regions, with region 1 centring onto the eyes, region 2 focussing on the mouth, and regions 3 and 4 centring on the two bars that were flanking the face to the right and to the left (see **Figure 2**). We found that as per task instructions, participants started each trial by first looking at the eye region (region 1, in on average 96% of the trials) before then moving onto the target region (regions 3 or 4). We also discovered that participants made a saccade only in on average 35% of the trials, and in some cases only after the trial had ended. This suggests that participants did not need to actively disengage from the central fixation cross in order to perform in the task, a finding which suggests that saccade-based effects may represent a limited source of information for attention biases in the Overlap task.

The average looking time for region 1 was input into two-way repeated measures ANOVA with the within-subject factors “expression” (fearful, neutral, happy) and trial type (Go, NoGo trials). The main effects of expression or trial type were not significant (Expression: [*F*(2,40) = 2.18, *p* = 0.13, $\eta_{\text{p}}^{2}$ = 0.10]; Trial type: [*F*(1,20) = 0.55, *p* = 0.47, $\eta_{\text{p}}^{2}$ = 0.03]) and neither were the interactions between expression × trial type ([*F*(2,40) = 0.12, *p* = 0.91, $\eta_{\text{p}}^{2}$ = 0.01; *BF01* = 21.0]).

We also repeated this analysis in a second step by including the individual trait anxiety scores as a covariate. Again, we found no significant main effect for either of the two factors, nor a significant interaction between the two factors when trait was included as a covariate (Expression: [*F*(2,38) = 0.23, *p* = 0.97, $\eta_{\text{p}}^{2}$ = 0.01]; Trial type: [*F*(1,19) = 1.24, *p* = 0.15, $\eta_{\text{p}}^{2}$ = 0.10]; Expression × Trial type: [*F*(2,38) = 0.35, *p* = 0.96, $\eta_{\text{p}}^{2}$ = 0.02; *BF01* = 21.3]). This suggests that the emotional expression displayed in the face stimulus did not affect the average looking time, and further, that is was not modulated by trait anxiety levels.

### Pupil Dilation Effects

**FIGURE 3:**
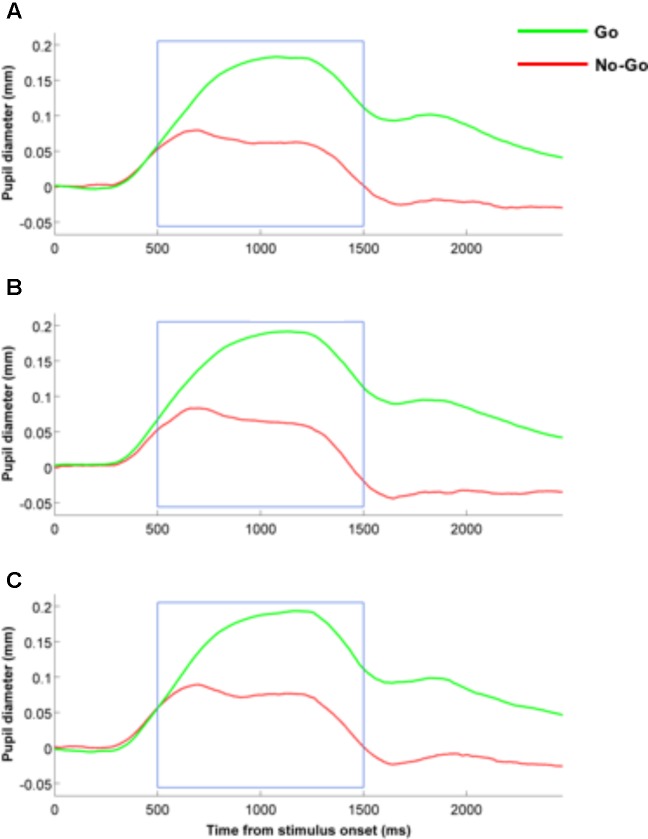
Time course of pupil dilation measures in both Go and No-Go conditions for fearful **(A)**, happy **(B)** and neutral **(C)** facial expressions.

Mean pupil dilation measures were input into two-way repeated measures ANOVA with the within-subject factors “expression” (fearful, neutral, happy) and trial type (Go, NoGo trials). The main effect of expression was not significant ([*F*(2,42) = 1.64, *p* = 0.21, $\eta_{\text{p}}^{2}$ = 0.07]) and neither were the interactions between expression x trial type ([*F*(2,42) = 0.79, *p* = 0.46, $\eta_{\text{p}}^{2}$ = 0.04]; *BF01* = 16.8). Only the main effect of trial type was significant ([*F*(1,21) = 82.2, *p* < 0.001, $\eta_{\text{p}}^{2}$ = 0.797]), with participants exhibiting increase pupil dilation in the Go trials (Mean = 0.161 mm), as opposed to the No-Go trials (Mean = 0.065 mm) (see also **Figure 3** for the pupil dilation time courses).

We also repeated this analysis in a second step by including the individual trait anxiety scores as a covariate. Again, when trait anxiety was included as a covariate, we found no significant main effect for either of the two factors (expression: [*F*(2,42) = 2.31, *p* = 0.11, $\eta_{\text{p}}^{2}$ = 0.10]; trial type: [*F*(1,21) = 1.95, *p* = 0.018, $\eta_{\text{p}}^{2}$ = 0.0.09]), nor a significant interaction between the two factors ([*F*(2,42) = 2.58, *p* = 0.09, $\eta_{\text{p}}^{2}$ = 0.11; *BF01* = 5.9]).

## Discussion

The current study combined the emotional go-no go Overlap task with eye-tracking techniques to investigate whether looking times and pupil dilations would vary as a function of emotional expression (hypothesis 1) and whether these effects would be moderated by individual differences in trait anxiety (hypothesis 2).

With regards to the first hypothesis, we did not find any expression-related differences in any of the eye-tracking measures, nor for RTs. This was supported using two analysis approaches, i.e., traditional *p*-value significance testing, as well as Bayesian hypothesis testing (Wagenmakers, 2007; Masson, 2011; Jarosz and Wiley, 2014). We did find though that participants were less accurate in neutral expression conditions, a finding that runs in line with research showing that proficiency with processing neutral expressions, or rather the lack of an expression exhibits protracted development into late childhood and early adolescence (Batty and Taylor, 2006; Durand et al., 2007; Thomas et al., 2007).

When we assessed the effect of trait anxiety differences, we again found no differences in the overt behavioral processing or the eye-tracking indices (looking time and pupil dilation patterns) and these null results were further supported by our Bayesian hypothesis testing. Together, these results suggest that at the commonly used presentation time of 500 ms in Overlap tasks or dot-probe paradigms (Bar-Haim, 2010), sub-clinically anxious adolescents will not exhibit attention biases away or toward a threat-relevant stimulus. The results replicate earlier findings by Cohen Kadosh et al. (2014), which tested a sample of younger (11–12 years) and older participants (17–18 years) with the same paradigm. In that study, the group found no behavioral effects in the older group, whereas the younger participants exhibited slower RTs for fearful faces. One rationale for further exploring this age group with eye-tracking methods was to assess whether, in the absence of an overt behavioral effect, there would nevertheless be covert effects that could be uncovered with a subtler technique (Just and Carpenter, 1976)^4^.

As laid out in **Tables 1A,B**, there is some evidence that clinically anxious children and adolescents avoid threat-relevant stimuli (Gamble and Rapee, 2009; Shechner et al., 2015), yet it is unclear whether similar attention biases would also characterize subclinically anxious participants. The current study can help address this question by showing that as found for low-anxious children and adolescents (Shechner et al., 2013), high (but not clinically) anxious adolescents do not exhibit biases either toward or away from threat-relevant stimuli. Given that high anxiety levels and difficulties with emotion regulation posit a risk for developing a psychiatric disorder (Kessler et al., 2007; Keshavan et al., 2014), our results suggest that the underlying cognitive biases may not simply linearly increase along with anxiety levels, but rather, that the underlying processing mechanisms differ between subclinically anxious and clinically anxious adolescents. We note though that this interpretation is based on previous studies that reported attention biases in clinically anxious participants (Bar-Haim, 2010) as the current sample did not include a group of clinically anxious adolescents. This also means that attention biases may not be a reliable index of atypical threat processing in subclinical samples.

Future research is now needed to fill this gap in our understanding how threat processing differs in subclinical vs. clinical groups of anxious youth. One possible way forward might be to systematically explore the effect of stimulus duration, as some research has shown that shorter and longer duration than 500 ms affects the direction of the observed attention biases (Bar-Haim, 2010). In addition, as the current study used the Overlap task rather than a traditional dot-probe paradigm, it would be important to assess the task-specificity of the current results by comparing both paradigms side by side in the same participants with varying anxiety levels. Similarly, the effects of other factors, such as attention control for non-emotional stimuli could be explored. Last, the use of more complex, contextualized paradigms might be another way forward, such as using faces within social scenes to explore interpretation biases, rather than low level attention biases alone. Eye-tracking measures should prove to be an excellent technique to explore all these factors, as they can provide some additional insights into looking times and distributions across the visual display – and the time-course and direction of the bias. Moreover, variations in pupil dilation could add information on intrinsic responses to emotional content.

In recent years, there have been some encouraging clinical trials using attention bias modification paradigms (Bar-Haim, 2010), such as the dot-probe task, to reduce anxiety clinically anxious children and adolescents (Bar-Haim et al., 2011; Eldar et al., 2011). The elegance of using a dot-probe paradigm is that it is simple to use and could even be administered remotely. This is all the more compelling given that current frontline treatments, such as cognitive behavioral therapy are costly to administer, often requiring face-to-face therapist time. And indeed, mobile app development has already started to plug this perceived gap in the market with a range of downloadable apps aimed at modifying cognitive biases (BMI, 2016). We note though that more research is urgently needed to provide a solid scientific foundation to these mobile intervention approaches.

## Conclusion

In this study, we investigated attentional biases in a subclinical sample of subclinically anxious adolescents. Using both an overt behavioral attention reorienting response in the Overlap task as well as eye-tracking techniques, we were unable to find evidence of a significant bias toward threatening stimuli. The results can help close a gap in the current literature by showing that much like low-anxious adolescents, subclinically anxious adolescents exhibit no attentional biases in the Overlap task. Together with previous research findings in clinically anxious participants which have reported high levels of attention biases, our results seem to suggest that, attention biases do no increase linearly as a function of individual anxiety level. Future research is now needed to explore the contribution of additional factors, such as depression for example, or of less automatic biases than those measured by dot-probe or overlap paradigms, such as interpretation biases engaged by more complex social stimuli.

## Ethics Statement

This study was carried out in accordance with the recommendations of research guidelines for conducting experiments with human subjects, Medical Science Division, University of Oxford. The protocol was approved by the University of Oxford Research Ethics Committee. All subjects gave written informed consent in accordance with the Declaration of Helsinki.

## Author Contributions

KCK and JL designed the task. SH, LS, and MD acquired the behavioral and eye-tracking data. KCK, SH, LS, and MD analyzed the data and all the authors contributed to writing the manuscript.

## Conflict of Interest Statement

The authors declare that the research was conducted in the absence of any commercial or financial relationships that could be construed as a potential conflict of interest.

## Footnotes

1 Note that in order to control for variations based on sex, hormones or brain maturation, only girls were tested research (Goddings et al., 2012).

2 www.mindgarden.com

3 http://psychtoolbox.org

4 We note that the sample tested in the current study has a lower average age and slightly wider age-range than the older participant group (i.e., 15.4 years vs. 17.0 years) in the Cohen Kadosh et al. (2014), study yet additional analysis on the current sample using a restricted age range did not change the results (all *p*s > 0.156).
